# RAAS inhibitors directly reduce diabetes‐induced renal fibrosis via growth factor inhibition

**DOI:** 10.1113/JP277002

**Published:** 2018-11-02

**Authors:** Sandor Koszegi, Agnes Molnar, Lilla Lenart, Judit Hodrea, Dora Bianka Balogh, Tamas Lakat, Edgar Szkibinszkij, Adam Hosszu, Nadja Sparding, Federica Genovese, Laszlo Wagner, Adam Vannay, Attila J Szabo, Andrea Fekete

**Affiliations:** ^1^ MTA‐SE “Lendület” Diabetes Research Group Hungarian Academy of Sciences and Semmelweis University Budapest Hungary; ^2^ 1st Department of Paediatrics Semmelweis University Budapest Hungary; ^3^ Department of Transplantation and Surgery Semmelweis University Budapest Hungary; ^4^ Nordic Bioscience Biomarkers & Research Herlev Denmark; ^5^ Biomedical Sciences Faculty of Health and Medical Science University of Copenhagen Copenhagen Denmark; ^6^ MTA‐SE Paediatrics and Nephrology Research Group Hungarian Academy of Sciences and Semmelweis University Budapest Hungary

**Keywords:** diabetic nephropathy, renin‐angiotensin‐aldosterone system inhibitors, tubulointerstitial fibrosis, profibrotic growth factors, PDGF, CTGF

## Abstract

**Key points:**

- Increased activation of the renin‐angiotensin‐aldosterone system (RAAS) and elevated growth factor production are of crucial importance in the development of renal fibrosis leading to diabetic kidney disease.
- The aim of this study was to provide evidence for the antifibrotic potential of RAAS inhibitor (RAASi) treatment and to explore the exact mechanism of this protective effect.
- We found that RAASi ameliorate diabetes‐induced renal interstitial fibrosis and decrease profibrotic growth factor production.
- RAASi prevents fibrosis by acting directly on proximal tubular cells, and inhibits hyperglycaemia‐induced growth factor production and thereby fibroblast activation.
- These results suggest a novel therapeutic indication and potential of RAASi in the treatment of renal fibrosis.

**Abstract:**

In diabetic kidney disease (DKD) increased activation of renin‐angiotensin‐aldosterone system (RAAS) contributes to renal fibrosis. Although RAAS inhibitors (RAASi) are the gold standard therapy in DKD, the mechanism of their antifibrotic effect is not yet clarified. Here we tested the antifibrotic and renoprotective action of RAASi in a rat model of streptozotocin‐induced DKD. *In vitro* studies on proximal tubular cells and renal fibroblasts were also performed to further clarify the signal transduction pathways that are directly altered by hyperglycaemia. After 5 weeks of diabetes, male Wistar rats were treated for two more weeks *per os* with the RAASi ramipril, losartan, spironolactone or eplerenone. Proximal tubular cells were cultured in normal or high glucose (HG) medium and treated with RAASi. Platelet‐derived growth factor (PDGF) or connective tissue growth factor (CTGF/CCN2)‐induced renal fibroblasts were also treated with various RAASi. In diabetic rats, reduced renal function and interstitial fibrosis were ameliorated and elevated renal profibrotic factors (TGFβ1, PDGF, CTGF/CCN2, MMP2, TIMP1) and alpha‐smooth muscle actin (αSMA) levels were decreased by RAASi. HG increased growth factor production of HK‐2 cells, which in turn induced activation and αSMA production of fibroblasts. RAASi decreased tubular PDGF and CTGF expression and reduced production of extracellular matrix (ECM) components in fibroblasts. In proximal tubular cells, hyperglycaemia‐induced growth factor production increased renal fibroblast transformation, contributing to the development of fibrosis. RAASi, even in non‐antihypertensive doses, decreased the production of profibrotic factors and directly prevented fibroblast activation. All these findings suggest a novel therapeutic role for RAASi in the treatment of renal fibrosis.

## Key points

- Increased activation of the renin‐angiotensin‐aldosterone system (RAAS) and elevated growth factor production are of crucial importance in the development of renal fibrosis leading to diabetic kidney disease.
- The aim of this study was to provide evidence for the antifibrotic potential of RAAS inhibitor (RAASi) treatment and to explore the exact mechanism of this protective effect.
- We found that RAASi ameliorate diabetes‐induced renal interstitial fibrosis and decrease profibrotic growth factor production.
- RAASi prevents fibrosis by acting directly on proximal tubular cells, and inhibits hyperglycaemia‐induced growth factor production and thereby fibroblast activation.
- These results suggest a novel therapeutic indication and potential of RAASi in the treatment of renal fibrosis.

## Introduction

Diabetes mellitus (DM) is a major health concern, impairing the quality of life and diminishing the life expectancy of millions of people (International Diabetes Federation, 2017). Diabetic kidney disease (DKD) affects more than 20% of all diabetic patients, and due to limited therapeutic options it remains the leading cause of chronic kidney disease (CKD) (Saran *et al*. 2018). Current treatment strategies may hinder kidney injury, but are unable to stop the progression into CKD (Solis‐Herrera *et al*. 2014). Therefore, a better understanding of the molecular mechanisms involved in the pathophysiology of DKD is needed in the research for novel therapeutic concepts.

The progression of DKD is caused by haemodynamic and metabolic changes leading to structural and functional alterations. Structural changes, starting with glomerular basement membrane thickening and mesangial matrix expansion, gradually progress into glomerulosclerosis and interstitial fibrosis, and result in glomerular hyperfiltration and microalbuminuria (Tervaert *et al*. 2010). Chronic and uncontrolled hyperglycaemia increases the production of several profibrotic factors such as transforming growth factor β (TGFβ), platelet‐derived growth factor (PDGF), and connective tissue growth factor/CCN family (cysteine‐rich 61, connective tissue growth factor, nephroblastoma overexpressed) member 2 (CTGF/CCN2), and contributes to fibrotic connective tissue accumulation characterised by elevated fibronectin and collagen levels (Garud & Kulkarni, 2014). Fibroblasts are the main effector cells of fibrosis and their activation is indicated by alpha‐smooth muscle actin (αSMA) production. When activated, they serve as primary collagen‐producing cells and contribute to extracellular matrix (ECM) accumulation (Wynn, 2008). In this state the balance of ECM dynamics is disturbed as the rate of production exceeds the rate of degradation and therefore ECM components such as type I and type III collagen, fibronectin and matrix metalloproteinases (MMPs) accumulate (Kolset *et al*. 2012).

The formation and degradation of ECM components lead to specific pro‐collagen terminal fragments and protein neo‐epitopes secreted into urine (Genovese *et al*. 2014), which have recently been discovered as novel, early urinary biomarkers of renal fibrosis. Two of them are neo‐epitopes of MMP‐9‐mediated degradation of type III collagen (C3M) and type IV collagen alpha 3 chain known as tumstatin (TUM) (Genovese *et al*. 2016; Nielsen *et al*. 2018). Numerous recent studies have confirmed the association of urinary C3M (uC3M) with renal fibrosis in different rat models including 5/6 Nx, anti‐Thy 1.1, adenine nephropathy and type 2 diabetic nephropathy models (Papasotiriou *et al*. 2015; Dower *et al*. 2017). In a proteinuria‐driven rat model, uC3M levels were elevated and measurable even before being histologically detectable (Hijmans *et al*. 2017). In type 1 diabetes these markers have not been tested yet and the effect of RAASi treatment on their excretion has not been investigated either.

Until recently glomeruli were considered to be the primary sites of DKD, but an increasing body of evidence shows that hyperglycaemia‐induced tubulointerstitial lesions have a prominent causative role as well. Hyperglycaemia increases tubular glucose load and leads to tubular hyperplasia and hypertrophy through early functional changes like primary proximal tubular hyperreabsorption. Increased DNA synthesis induces the transition to senescence where local pro‐inflammatory cytokine release, growth factor production and ECM components form a basis of tubulointerstitial fibrosis (Vallon, 2011). Available therapeutic strategies are inefficient in preventing the progression of kidney injury (Karihaloo, 2012), and therefore novel treatment options are definitely needed.

Several studies demonstrate that various RAASi effectively decrease renal functional and structural damage in DM (Mifsud *et al*. 2001; Osicka *et al*. 2001; Gellai *et al*. 2016). Some of these highlight the exceptional role of aldosterone antagonists (Taira *et al*. 2008; Banki *et al*. 2012), although the molecular pathways involved in their renoprotective effect are still not fully understood.

The aim of the present study was to understand the underlying mechanisms involved in DM‐induced renal fibrosis and to investigate the potential antifibrotic effect of RAASi. Furthermore, the effects of RAASi on proximal tubular epithelial cells and on renal fibroblasts were studied to explore their direct renoprotective potential.

## Methods

### Ethical approval

All animal procedures were approved by the Committee on the Care and Use of Laboratory Animals of the Council on Animal Care at Semmelweis University, Budapest, Hungary (PEI/001/380‐4/2013) and conform to the principles and regulations of *The Journal of Physiology*. The investigators state their confirmation of compliance with the ethical principles under which *The Journal of Physiology* operates and that our work complies with its animal ethics checklist.

### Materials

All chemicals were purchased from Sigma‐Aldrich (Darmstadt, Germany) unless stated otherwise, and all standard plastic laboratory equipment was purchased from Sarstedt (Nümbrecht, Germany).

### Animals, induction of diabetes and experimental groups

Six‐week‐old male Wistar rats (*Rattus norvegicus*, RGD Cat. No. 13508588, RRID:RGD_13508588) were purchased from “Toxi‐Coop” Toxicological Research Centre (Dunakeszi, Hungary) and kept in plastic cages under 12‐hour dark/light cycle at constant temperature (24 ± 0.2°C) with *ad libitum* access to standard rodent chow and drinking water. Diabetes was induced with a single i.p. injection of 65 mg (kg body wt)^−1^ streptozotocin (STZ), dissolved in 0.1 M citrate buffer (pH 4.5). Blood glucose levels were measured three times from the tail vein after an overnight fast with a D‐Cont IDEAL device (77 Elektronika, Budapest, Hungary). Animals were considered diabetic if peripheral blood glucose level was above 15 mmol L^−1^ 72 h after the STZ injection and remained elevated. Five weeks after the induction of diabetes rats were randomised into five groups (*n* = 7–8 animals/group) and were treated daily by oral gavage for 2 weeks either with: (i) isotonic saline as vehicle (D); or (ii) ramipril (D + ramipril, 10 μg (kg body wt)^−1^ day^−1^); losartan (D + losartan, 20 mg (kg body wt)^−1^ day^−1^); spironolactone (D + spironolactone, 50 mg (kg body wt)^−1^ day^−1^) or eplerenone (D + eplerenone, 50 mg (kg body wt)^−1^ day^−1^). Doses were adopted from our previous studies in line with literary data where effective blockade of angiotensin‐converting enzyme (ACE), angiotensin II receptor 1 (AT1) or aldosterone activity was achieved without changes in systemic blood pressure (Taira *et al*. 2008; Failli *et al*. 2009; Banki *et al*. 2012). Non‐diabetic, age‐matched animals (control; *n* = 8 animals/group) received equivalent volumes of citrate buffer without STZ once, and the same amount of saline by oral gavage daily, at the same time as the diabetic animals throughout the 2‐week treatment period. At the end of the experimental protocol rats were anaesthetised by a mixture of 75 mg (kg body wt)^−1^ ketamine (Richter Gedeon, Budapest, Hungary) and 10 mg (kg body wt)^−1^ xylazine (Medicus Partner, Biatorbagy, Hungary) administered i.p., after which breathing and cardiac function decreased and terminal blood was drawn from the abdominal aorta to kill the animals. Blood, urine and kidney samples were collected and stored for further investigations.

### Arterial blood pressure

Systolic and diastolic blood pressures were measured on the tail vein and mean arterial pressure was calculated using a CODA tail cuff standard monitoring system (EMKA Technologies, Paris, France), which uses proprietary volume pressure recording, a clinically validated technology (Kurtz *et al*. 2005).

### Metabolic and renal parameters

Body and kidney weights were measured, and kidney‐to‐body weight ratio and creatinine clearance were calculated. Serum metabolic (glucose, fructosamine) and renal (blood urea nitrogen, creatinine) parameters were determined with commercially available kits on a Hitachi 912 chemistry analyser (Roche Hitachi, Basel, Switzerland).

### Renal histology and morphometric analysis

Kidneys were fixed in 4% buffered formalin, embedded in paraffin and 5 μm thick sections were taken.

Glomerular hypertrophy was determined in periodic acid‐Schiff (PAS)‐stained sections as previously described (Degrell *et al*. 2009) by measuring 20 glomerular tuft areas at 400× magnification from each animal, excluding incomplete glomeruli along the sample edge. The number of pixels containing purple mesangial matrix deposition was divided by the total number of pixels in the glomerulus to obtain the percentage area of mesangial matrix deposition.

Tubulointerstitial fibrosis was evaluated in Masson's trichrome‐stained sections and Picrosirius Red staining was performed for supplemental fibrosis. Ten areas at 200× (Masson's) or 100× (Picrosirius) magnification from each kidney cross‐section were randomly selected and specifically stained interstitial areas were measured. The number of pixels containing stained fibrotic tissue was divided by the total number of pixels in the area to obtain the percentage of tubulointerstitial fibrosis and collagen deposition.

Areas were measured with Panoramic Viewer software version 1.15.2 (3DHISTECH, RRID: SCR_016296). Analysis was performed in a double blinded fashion with computer‐assisted morphometry using Adobe Photoshop CS6 (Adobe Systems, RRID:SCR_014199) and Scion Image software version 1.49 (National Institutes of Health, RRID:SCR_008673).

### Cell cultures and treatments

Human kidney 2 proximal tubular epithelial (HK‐2) cells (LGC Standards, ATCC Cat. No. CRL‐2190, RRID:CVCL_0302) were cultured in DMEM containing 5.5 mM glucose (Gibco, supplied by Life Technologies, Carlsbad, CA, USA) and normal rat kidney fibroblast (NRK‐49F) cells (LGC Standards, ATCC Cat. No. CRL‐1570, RRID:CVCL_2144) were maintained in DMEM containing 25 mM glucose (Gibco), both supplemented with 10% FBS (Gibco), 1% penicillin/streptomycin and 1% L‐glutamine, and incubated at 5% CO_2_ and 37°C. Before the treatments, HK‐2 and NRK‐49F cells were plated in 6‐well plates (5 × 10^5^ cells/well) for 24 h in serum‐free medium. In the case of HK‐2 cells, the medium was changed and cells were kept under normal (control; 5.5 mM) or high glucose (HG; 35 mM) conditions for 24 h. HG groups were treated with either 10 μM ramipril (HG + ramipril), or 10 μM losartan (HG + losartan), or 200 nM spironolactone (HG + spironolactone), or 10 μM eplerenone (HG + eplerenone). Control cells were treated with vehicle (DMSO) alone (*n* = 6 wells/group). Drug doses were adopted from the literature (Oroszlan *et al*. 2010; Wei *et al*. 2013; Xiao *et al*. 2016) and their non‐toxic properties were tested by viability assays (data not shown).

NRK‐49F cells were treated with PDGF (10 ng mL^−1^) or CTGF/CCN2 (10 ng mL^−1^) and with various RAASi as described above. In pilot studies the most effective proliferative doses were determined from different PDGF and CTGF/CCN2 concentration series by proliferative assays (data not shown). Cells were subsequently incubated at 5% CO_2_ and 37°C, detached with 0.25% trypsin‐EDTA (Gibco) and lysed with the same buffers that were used for kidney homogenates.

To differentiate between the effect of direct glucotoxicity and the osmotic effect of high glucose (HG), HK‐2 cells were cultured in normal (5.5 mM) or HG (35 mM) media, as well as in isosmotic control mannitol (5.5 mM glucose + 29.5 mM mannitol).

### Biomarkers of ECM formation and degradation

The biomarkers uC3M (measuring collagen type III fragment degraded by MMP‐9), rPRO‐C3 (measuring type III collagen formation) and TUM (measuring tumstatin, a collagen type IV fragment degraded by MMP‐9) were measured in rat urine samples. FBN‐C (measuring C‐terminal of fibronectin turnover) and PRO‐C4 (measuring type IV collagen formation) were measured in HK‐2 and NRK‐49F cell supernatant samples, using competitive enzyme‐linked immunosorbent assays (ELISAs) developed by Nordic Bioscience (Herlev, Denmark). To normalize for urine output, levels were divided by urinary creatinine levels measured with the QuantiChromTM Creatinine kit (BioAssay Systems). The assays were carried out at Nordic Bioscience laboratories following previously described protocols (Leeming *et al*. 2013; Nielsen *et al*. 2013, 2018; Bager *et al*. 2016; Genovese *et al*. 2016). Briefly, astreptavidin‐coated 96‐well ELISA plates (Roche, Cat. No. 11940279) were coated with 100 μL biotinylated peptide for 30 min at 20°C. Plates were washed five times in washing buffer followed by incubation with 20 μL standard peptide or sample together with HRP‐conjugated monoclonal antibody and incubated for 20 h at 4°C (uC3M, rPRO‐C3, FBN‐C) or 1 h at 20°C (TUM, PRO‐C4). Plates were washed five times followed by incubation with 100 μL 3,3ʹ,5,5‐tetramethylbenzidine (Kem‐En‐Tec, Cat. No. 4380H, Taastrup, Denmark) for 15 min at 20°C in the dark. To stop the reaction, 1% sulfuric acid solution was added, and plates were analysed with the ELISA reader at 450 nM, with 650 nm as reference (VersaMax, Molecular Devices, CA, USA).

### Fluorescent immunohistochemistry

Frozen kidney sections were embedded in Shandon cryomatrix (Thermo Fisher Scientific, Waltham, MA, USA) and cut into 10‐μm slices with a cryostat. NRK‐49F cells were cultured in tissue culture chambers. After repeated washes with PBS, cells were fixed in 4% formalin, washed again, and permeabilised with Triton X‐100. Samples were incubated for 2 h with specific primary antibodies: anti‐αSMA (Sigma‐Aldrich, Cat. No. A2547, RRID:AB_476701) diluted 1:1000 or anti‐PDGFR‐β (Santa Cruz Biotechnology, Cat. No. sc‐432, RRID:AB_631068) diluted 1:100. After repeated washes, slides were incubated with specific secondary antibodies: goat anti‐mouse IgG Alexa fluor 488 (Thermo Fisher Scientific, Cat. No. A‐11001, RRID:AB_2534069) diluted 1:100 or goat anti‐rabbit Alexa fluor 568 (Thermo Fisher Scientific, Cat. No. A‐11036, RRID:AB_10563566) diluted 1:100. F‐actin was immunostained by incubation with phalloidin‐TRITC diluted 1:300 for 1 h at room temperature. Samples were counterstained with Hoechst 33342 (Cell Signaling Technology, Cat. No. 4082S, RRID:AB_10626776) diluted 1:1000 to visualise nuclei. Appropriate controls were prepared omitting the primary antibody to assure specificity and to avoid autofluorescence. After being dried, sections were fixed with Vectashield Mounting Medium (Vector Laboratories, Cat. No. H‐1000, RRID:AB_2336789). Coverslipped slides were analysed with a Zeiss LSM 510 Meta confocal laser scanning microscope (LSM Image Examiner, RRID:SCR_014344) with 63× or 100× magnification objectives or, for phalloidin‐stained slides, with an Olympus IX81 fluorescence microscope at 100× magnification.

### Western blot analysis

Kidney cortex samples or cells were homogenised in lysis buffer (1 M Tris, 0.5 M EGTA, 1% Triton X‐100, 0.25 M NaF, 0.5 M phenylmethylsulfonyl fluoride, 0.5 M sodium orthovanadate, 5 mg mL^−1^ leupeptin, and 1.7 mg mL^−1^ aprotinin, pH 7.4). Lysates were centrifuged at 13,000 rpm, at 4°C for 10 min. Protein concentration of the supernatants was measured with a detergent‐compatible protein assay kit (Bio‐Rad Hungary, Budapest, Hungary). Solubilised samples were electrophoretically resolved: appropriate amounts (25 μg for tissue and 18 μg for cells) of protein were loaded onto 4–20% gradient Mini‐PROTEAN TGX polyacrylamide precast gels (Bio‐Rad Hungary) and transferred to nitrocellulose membranes. Membranes were blocked in 5% w/v non‐fat dried milk in Tris‐buffered saline (TBS) for 1 h at room temperature and incubated with primary antibody anti‐αSMA (Sigma‐Aldrich, Cat. No. A2547, RRID:AB_476701) diluted 1:2000 at 4°C overnight. After washing with 1% w/v non‐fat dried milk in TBS‐Tween, membranes were incubated with the appropriate HRP‐conjugated secondary antibody goat anti‐mouse IgG (Cell Signaling Technology, Cat. No. 7076, RRID:AB_330924) diluted 1:6000. Antibody‐antigen complexes were detected using enhanced chemiluminescence (ECL by GE Healthcare Life Sciences, Budapest, Hungary) and immunoreactive bands were quantified densitometrically on Versadoc, Quantity One 1‐D Analysis software (Bio‐Rad Hungary, RRID:SCR_01428) as integrated optical density (IOD) after subtraction of background. The IOD was factored for Ponceau S staining to correct for any variations in total protein loading. The protein abundance was represented as IOD/Ponceau S/Internal control.

### Reverse transcription polymerase chain reaction (RT‐PCR)

Total RNA was extracted using the RT050 Total RNA isolation Mini Kit (Geneaid Biotech, New Taipei City, Taiwan). The quality and quantity of isolated RNA was measured on a NanoDrop ND‐1000 spectrophotometer (Baylor College of Medicine, Houston, TX, USA). Five hundred nanograms each of *Tgfb1*, *Pdgfb*, *Ctgf*, *Mmp2*, *Timp1*, *Timp2* mRNA from rat kidney samples and 500 ng each of *TGFB1*, *PDGFB* and *CTGF* mRNA from human proximal tubular epithelial cells were reverse‐transcribed using a First Strand cDNA Synthesis Kit for RT‐PCR (Thermo Fisher Scientific). The expression of the mRNAs was determined in triplicate with 1 μL cDNA samples obtained by qPCR using 10 μL SYBR Green I Master enzyme mix (Roche Diagnostics, Mannheim, Germany) and 10 pmol μL^−1^ of each specific primer (Invitrogen, Budapest, Hungary), following sequences designed by Lasergene PrimerSelect software version 7.1.0 (DNASTAR, RRID:SCR_016295) based on nucleotide sequences from National Center for Biotechnology Information's nucleotide database (primer sequences: Table 1). Results were analysed by LightCycler 480 software version 1.5.0 (Roche Diagnostics, RRID:SCR_012155). The mRNA expression of interest was normalised against mRNA expression of 18S ribosomal RNA (*Rn18s* or *RN18S*) from the same samples as internal control.

**Table 1:** PCR primer sequences used in this study

Gene name	Regular name	NCBI ID	Primer pairs		Product lenght	*T*_a_
*Tgfb1*	Rat TGFβ1	21803	Forward:	5ʹ‐GCA CCG GAG AGC CCT GGA TAC C‐3ʹ	222 bp	60°C
			Reverse:	5ʹ‐CCC GGG TTG TGT TGG TTG TAG AGG‐3ʹ		
*Pdgfb*	Rat PDGFB	24628	Forward:	5ʹ‐TCG ATC GCA CCA ATG CCA ACT TCC‐3ʹ	236 bp	62°C
			Reverse:	5ʹ‐CAC GGG CCG AGG GGT CAC TAC TGT‐3ʹ		
*Ctgf*	Rat CTGF	64032	Forward:	5ʹ‐TCC ACC CGG GTT ACC AAT GAC AAT AC‐3ʹ	195 bp	58°C
			Reverse:	5ʹ‐CTT AGC CCG GTA GGT CTT CAC ACT GG‐3ʹ		
*Mmp2*	Rat MMP2	81686	Forward:	5ʹ‐TCC CCC AAA ACA GAC AAA GAG T‐3ʹ	224 bp	56°C
			Reverse:	5ʹ‐TTG CGG GGA AAG AAG TTG TAG T‐3ʹ		
*Timp1*	Rat TIMP1	116510	Forward:	5ʹ‐GGC GCC CTT TGC ATC TCT GG‐3ʹ	250 bp	58°C
			Reverse:	5ʹ‐GGC GAA CCG GAA ACC TGT GG‐3ʹ		
*Timp2*	Rat TIMP2	29543	Forward:	5ʹ‐GCC CTG GGA CAC GCT TAG CAT C‐3ʹ	220 bp	59°C
			Reverse:	5ʹ‐GTA CCA CGC GCA AGA ACC ATC ACT‐3ʹ		
*Rn18s*	Rat 18S ribosomal RNA	100861533	Forward:	5ʹ‐GCG GTC GGC GTC CCC CAA CTT CTT‐3ʹ	105 bp	60°C
			Reverse:	5ʹ‐GCG CGT GCA GCC CCG GAC ATC TA‐3ʹ		
*TGFB1*	Human TGFβ1	7040	Forward:	5ʹ‐GCG TGC GGC AGC TGT ACA TTG ACT‐3ʹ	174 bp	60°C
			Reverse:	5ʹ‐CGA AGG CGC CCG GGT TAT GC‐3ʹ		
*PDGFB*	Human PDGFB	5155	Forward:	5ʹ‐AGA TGG GGC CGA GTT GGA CCT GAA‐3ʹ	163 bp	62°C
			Reverse:	5ʹ‐GCG CCG GGA GAT CTC GAA CAC CT‐3ʹ		
*CTGF*	Human CTGF	1490	Forward:	5ʹ‐GTC CAC CCG GGT TAC CAA TGA CAA‐3ʹ	228 bp	58°C
			Reverse:	5ʹ‐CAG GAT CGG CCG TCG GTA CAT ACT‐3ʹ		
*RN18S*	Human 18S ribosomal RNA	HQ387008.1	Forward:	5ʹ‐CGC GGA TCC GAA CAC TGC GTT TGC TGG CTT TGA TG‐3ʹ	136 bp	60°C

*T*_a_, annealing temperature.

### Statistical analysis

To test the hypothesis that RAAS inhibition plays a renoprotective antifibrotic role in the rat model of STZ‐induced DKD, statistical analyses were performed using GraphPad Prism software version 6.01 (GraphPad Software, RRID:SCR_002798). To test if the values were from a Gaussian distribution, a Kolmogorov‐Smirnov normality test was performed. Data were analysed by one‐way ANOVA followed by Bonferroni's multiple‐comparison *post hoc* test for all parametrical comparisons, or in the case of non‐parametric data, by Kruskal‐Wallis ANOVA on ranks. Significance was set *a priori* at *P* < 0.05, corrected for multiple comparisons. Data are presented using box and whisker plots with means and 95% confidence intervals.

## Results

### RAAS inhibitors improve metabolic and renal functional parameters in STZ‐induced diabetic rats

**Figure 1:**
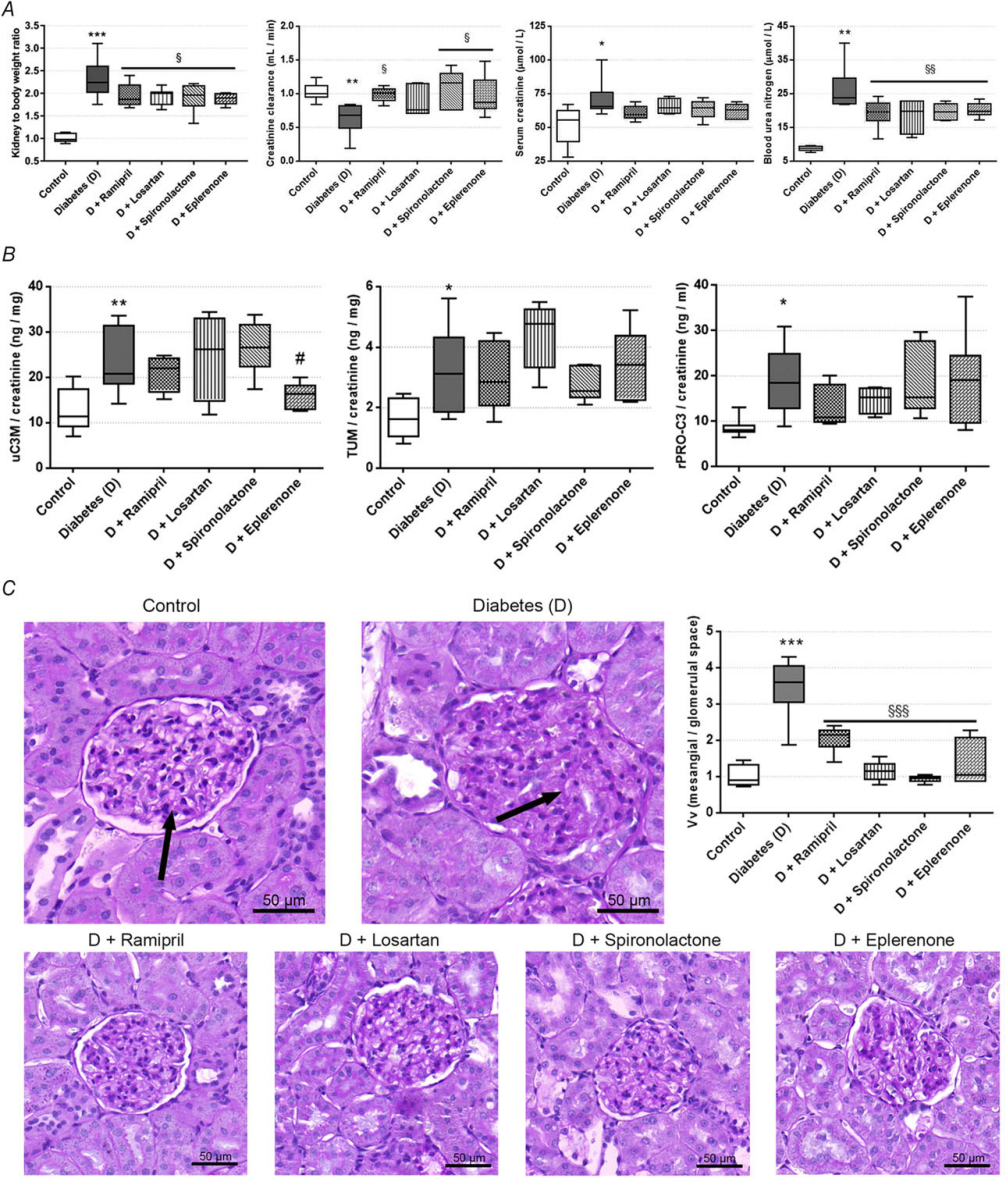
Renal functional parameters, kidney histology and urinary fibrotic markers of control, diabetic and renin‐angiotensin‐aldosterone system inhibitor‐treated diabetic rats *A*, kidney‐to‐body weight ratio, creatinine clearance, serum creatinine, and blood urea nitrogen were measured in rats. *B*, urinary collagen type III degradation fragment (uC3M), tumstatin (TUM) and N‐terminal pro‐peptide of rodent type III collagen (rPRO‐C3) levels measured from rat urine. *C*, representative periodic acid‐Schiff (PAS)‐stained histological sections and quantitative evaluation of glomerular hypertrophy and mesangial matrix expansion. Arrows mark mesangial matrix deposition stained with purple. 400× magnification; scale bar = 50 μm. Values are presented as means ± 95% confidence intervals; *n* = 7–8 rats/group; one‐way ANOVA followed by Bonferroniʹs multiple‐comparison *post hoc* test; ^*^ *P* < 0.05, ^**^ *P* < 0.01, ^***^ *P* < 0.001 *vs*. control; ^#^ *P* = 0.08, ^§^ *P* < 0.05, ^§§^ *P* < 0.01, ^§§§^ *P* < 0.001 *vs*. D.

Elevated serum glucose, fructosamine levels and lower body weights confirmed the development of type 1 diabetes mellitus. RAASi did not alter any of the metabolic parameters. Mean arterial pressure remained unaltered in all groups confirming that the examined effects of RAASi are independent of their antihypertensive properties (Table 2). A decline in renal functional parameters and higher kidney‐to‐body weight ratios indicate the development of DKD. RAASi reduced relative kidney enlargement, improved creatinine clearance and decreased renal retention parameters (Fig. 1 *A*). Urinary biomarkers of ECM remodelling showed increased collagen III formation (rPRO‐C3) and degradation (uC3M) and collagen IV turnover (TUM) in diabetic animals. Eplerenone treatment decreased the level of uC3M level (Fig. 1 *B*). Besides functional decline, a key histological features of DKD, massive glomerular hypertrophy and mesangial matrix expansion were observed in untreated diabetic rats. All of the RAASi minimised structural damage as reflected by smaller PAS positive glomerular areas (Fig. 1 *C*).

**Table 2:** Body weight, metabolic parameters and mean arterial pressure (MAP) of control, diabetic and renin‐angiotensin‐aldosterone system inhibitor‐treated diabetic rats

Parameters	Control	Diabetes (D)	D + ramipril	D + losartan	D + spironolactone	D + eplerenone
Body weight (g)	418 ± 23.4	276 ± 28.9*	265 ± 12.8*	258 ± 24.8*	245 ± 35.1*	288 ± 20.9*
Serum glucose (mmol L^−1^)	12.7 ± 1.15	47.3 ± 7.80*	44.1 ± 3.67*	43.8 ± 4.79*	42.6 ± 2.55*	34.8 ± 3.57
Serum fructosamine (μmol L^−1^)	153 ± 8.28	243 ± 16.3***	249 ± 10.6***	256 ± 17.6***	239 ± 11.5***	239 ± 16.5***
MAP (mmHg)	74.7 ± 7.51	73.9 ± 8.78	72.3 ± 5.46	73.8 ± 14.0	77.4 ± 16.0	77.3 ± 13.2

Values are presented as means ± 95% confidence limits; *n* = 7–8 rats/group; one‐way ANOVA followed by Bonferroniʹs multiple‐comparison *post hoc* test; ^*^ *P* < 0.05, ^***^ *P* < 0.001 *vs*. control.

### RAAS inhibitors ameliorate tubulointerstitial fibrosis in diabetic kidneys

**Figure 2:**
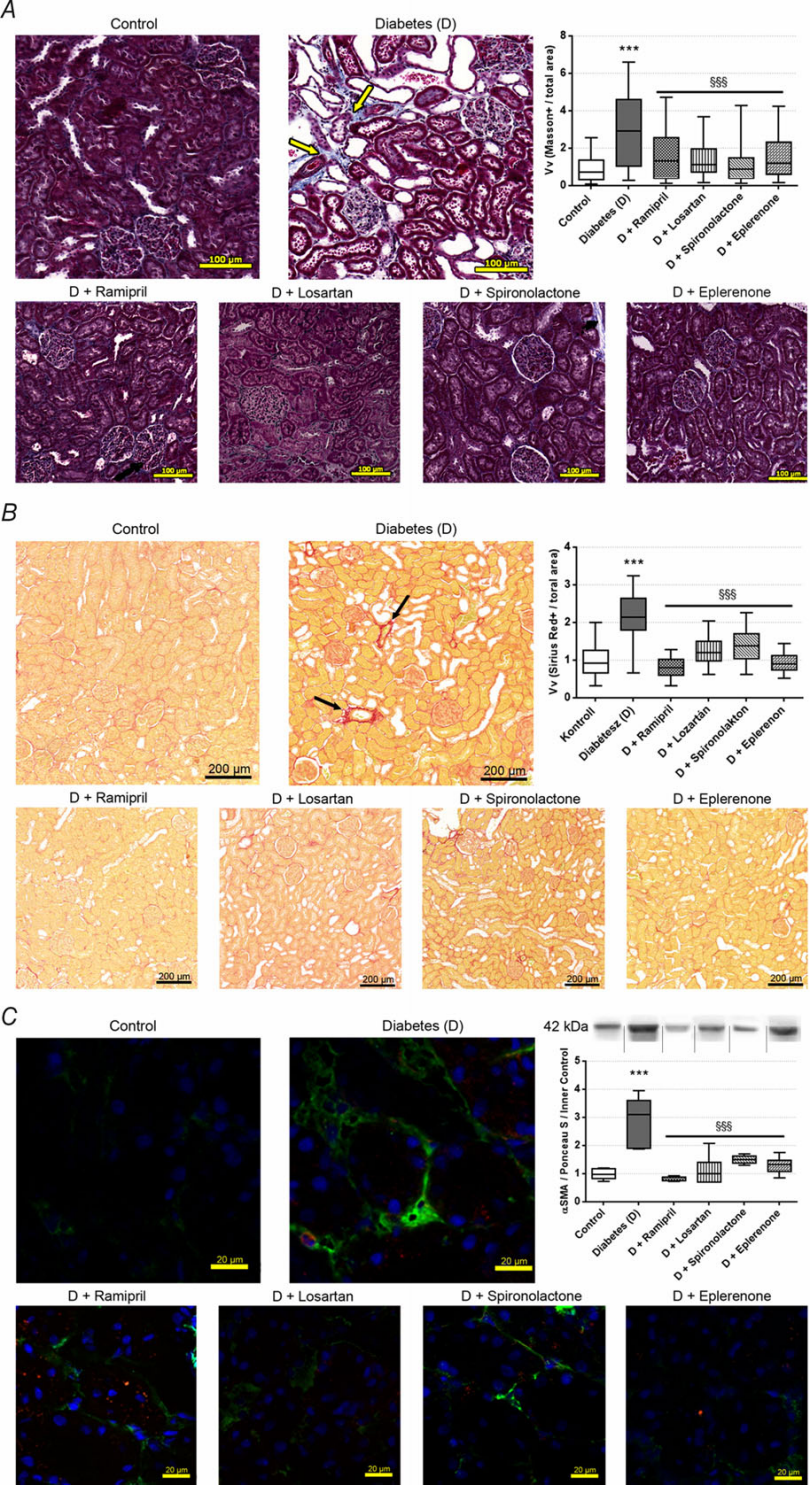
Fibrotic alterations in kidneys of control, diabetic and renin‐angiotensin‐aldosterone system inhibitor treated diabetic rats *A*, representative Massonʹs trichrome‐stained histological sections and quantitative evaluation of renal tubulointerstitial fibrosis by Masson‐positive and glomerulus‐free *vs*. total areas in the kidney cortex. Arrows mark fibrotic tissue stained with blue. 200× magnification; scale bar = 100 μm. *B*, representative Picrosirius Red‐stained histological kidney sections and quantitative evaluation of collagen accumulation. Arrows mark red‐stained collagen. 100× magnification; scale bar = 200 μm. On each graph values are presented as means ± 95% confidence intervals; *n* = 7–8 rats/group; Kruskal‐Wallis ANOVA on ranks; ^***^ *P* < 0.001 *vs*. control; ^§§§^ *P* < 0.001 *vs*. D. *C*, representative fluorescent immunohistochemical staining of renal sections and Western blot analysis of alpha‐smooth muscle actin (αSMA) protein levels in the kidneys, with representative examples above. Samples might be from different gels but were derived at the same time and processed in parallel. 630× magnification; green, αSMA; blue, nucleus; scale bar = 20 μm; values are presented as means ± 95% confidence intervals; *n* = 7–8 rats/group; one‐way ANOVA followed by Bonferroniʹs multiple‐comparison *post hoc* test; ^***^ *P* < 0.001 *vs*. control; ^§§§^ *P* < 0.001 *vs*. D.

Renal tubulointerstitial fibrosis was assessed on Masson's trichrome‐stained sections. The quantity of fibrotic connective tissue increased in diabetic animals, while all of the RAASi decreased tubulointerstitial fibrosis (Fig. 2 *A*). To assess accumulation of the collagen components of ECM, Picrosirius Red staining was performed, which showed increased amounts of collagen in diabetic kidneys. RAASi also significantly decreased renal collagen depositions (Fig. 2 *B*). Another widely accepted important and specific marker of fibrosis is the increased level of αSMA, which indicates the activation and differentiation of fibroblasts (Skalli *et al*. 1987). We demonstrated that in diabetic animals, the level of αSMA is significantly increased and the protein is mainly localised in the tubulointerstitial region. RAASi decreased the accumulation of αSMA protein (Fig. 2 *C*).

### Aldosterone antagonists decrease PDGF and CTGF/CCN2 levels in diabetic kidneys

**Figure 3:**
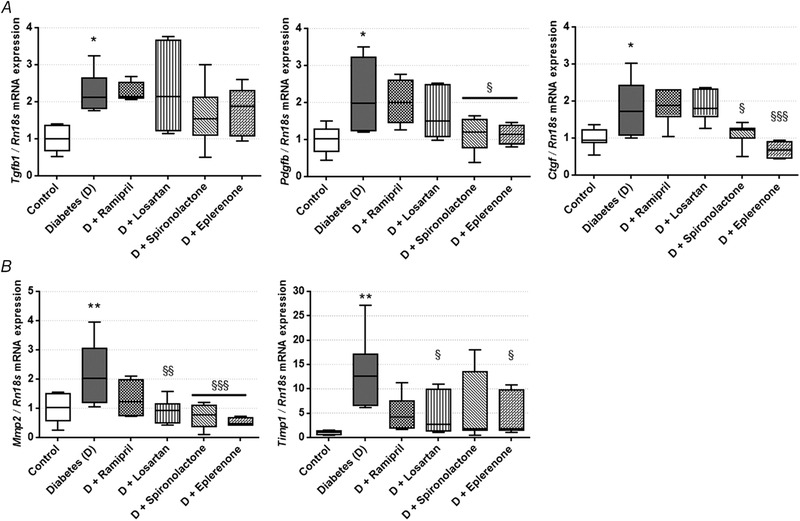
mRNA expression of profibrotic factors and fibrotic marker extracellular matrix enzymes in control, diabetic and renin‐angiotensin‐aldosterone system inhibitor‐treated diabetic rats *A*, transforming growth factor β 1 (*Tgfb1*), platelet‐derived growth factor B (*Pdgfb*) and connective tissue growth factor (*Ctgf*) mRNA expression measured in kidney. *B*, matrix metalloproteinase 2 (*Mmp2*) and tissue inhibitor of matrix metalloproteinase 1 (*Timp1*) mRNA expression measured in kidney. Values are presented as means ± 95% confidence intervals; *n* = 7–8 rats/group; one‐way ANOVA followed by Bonferroniʹs multiple‐comparison *post hoc* test; ^*^ *P* < 0.05, ^**^ *P* < 0.01 *vs*. control; ^§^ *P* < 0.05, ^§§^ *P* < 0.01, ^§§§^ *P* < 0.001 *vs*. D.

Since DKD‐associated cytokines induce fibrotic differentiation (Boor & Floege, 2011), we measured *Tgfb1*, *Pdgfb* and *Ctgf* mRNA expression in the renal cortex. In the diabetic group, expression of all profibrotic factors was elevated. RAASi did not alter *Tgfb1* expression, while aldosterone antagonists decreased *Pdgfb* and *Ctgf* expression (Fig. 3 *A*).

Matrix metalloproteinases facilitate ECM turnover and serve as a marker of renal fibrosis (Du *et al*. 2012). In diabetic rats renal *Mmp2* expression was increased, and aldosterone antagonists showed the most robust effect in minimizing *Mmp2* expression. Expression of the tissue inhibitor of matrix metalloproteinase 1 (*Timp1*) also increased in diabetes, following the increase in *Mmp2*. This change was also alleviated by losartan and eplerenone treatment (Fig. 3 *B*). The expression of *Timp2* and *Mmp9* remained the same in all groups (data not shown).

### RAAS inhibitors prevent PDGF and CTGF/CCN2 production in HK‐2 proximal tubular epithelial cells

**Figure 4:**
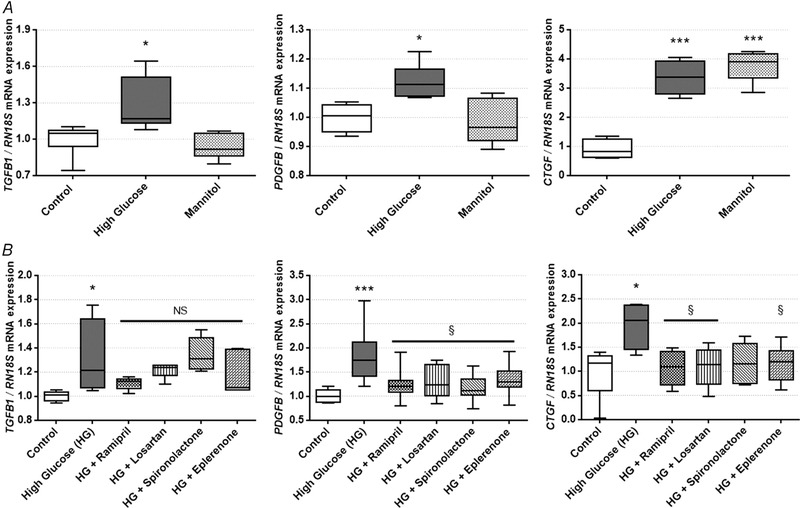
Profibrotic factor production in human kidney 2 (HK‐2) cells on normal, high glucose (HG), mannitol and renin‐angiotensin‐aldosterone system inhibitor (RAASi) treatment *A*, transforming growth factor β (*TGFB1*), platelet‐derived growth factor B (*PDGFB*), and connective tissue growth factor (*CTGF*) mRNA expression in HK‐2 proximal tubular epithelial cells on HG or osmotic control mannitol treatment. *B, TGFB1*, *PDGFB*, and *CTGF* mRNA expression in HK‐2 cells on HG and RAASi treatment. Values are presented as means ± 95% confidence intervals; *n* = 6 wells/group; one‐way ANOVA followed by Bonferroniʹs multiple‐comparison *post hoc* test; ^*^ *P* < 0.05, ^***^ *P* < 0.001 *vs*. control; ^§^ *P* < 0.05, NS, not significant *vs*. HG

*In vitro* experiments were performed to further clarify the profibrotic signal transduction pathways. HK‐2 cells were cultured both in normal, HG or mannitol‐treated isosmotic conditions to distinguish the *per se* effects of hyperosmolality and hyperglycaemia on proximal tubule cells. Expression of *TGFB1*, *PDGFB* and *CTGF* was increased in HG‐treated HK‐2 cells. *TGFB1* and *PDGFB* expression did not change with mannitol treatment, but, in contrast, mannitol enhanced *CTGF* mRNA expression. These results suggest that the elevation of *TGFB1* and *PDGFB* can be considered as a direct effect of glucotoxicity, while *CTGF* production is the result of the complex effect of hyperglycaemia and hyperosmolarity (Fig. 4 *A*).

RAASi significantly reduced elevated expression of both *PDGFB* and *CTGF* to the level of controls (Fig. 4 *B*). Since RAASi did not influence *TGFB1* expression either in the kidney or in HK‐2 cells, we focused our further analyses on *PDGFB* and *CTGF*.

### RAAS inhibitors restore normal fibronectin turnover in HK‐2 proximal tubular epithelial cells

**Figure 5:**
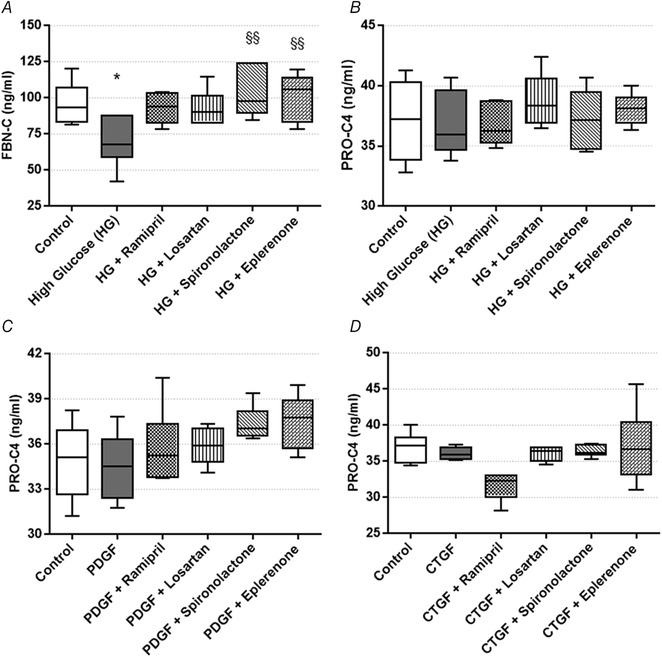
Extracellular matrix formation and degradation biomarkers measured in human kidney 2 (HK‐2) cells on high glucose (HG) and renin‐angiotensin‐aldosterone system inhibitor (RAASi) treatment and in normal rat kidney fibroblast (NRK‐49F) cells treated with profibrotic factors and RAASi *A*, C‐terminal of fibronectin (FBN‐C) turnover marker secretion by HK‐2 proximal tubular epithelial cells on HG and RAASi treatment. *B*, type IV collagen formation biomarker (PRO‐C4) secretion by HK‐2 cells on HG and RAASi treatment. *C* and *D*, PRO‐C4 secretion by NRK‐49F cells treated with platelet‐derived growth factor (PDGF; *C*) or connective tissue growth factor (CTGF; *D*). Values are presented as means ± 95% confidence intervals; *n* = 6 wells/group; one‐way ANOVA followed by Bonferroniʹs multiple‐comparison *post hoc* test; ^*^ *P* < 0.05 *vs*. control; ^§§^ *P* < 0.01 *vs*. HG.

The amount of C‐terminal of fibronectin (FBN‐C) secreted into the supernatant is a biomarker of fibronectin turnover. High glucose treatment reduced the level of FBN‐C (fibronectin turnover), which was restored to the level of controls by treatment with spironolactone and eplerenone (Fig. 5 *A*). Secreted FBN‐C levels in NRK cells were below the lower limit of detection. The amount of internal epitope in the 7S domain of type IV collagen (PRO‐C4) secreted into the cells’ supernatant serves as a collagen formation biomarker. No changes in type IV collagen formation in HK‐2 cells (Fig. 5 *B*) and NRK‐49F cells (Fig. 5 *C* and *D*) were detected in any of the goups.

### PDGF and CTGF/CCN2‐induced αSMA production of renal fibroblasts is prevented by RAAS inhibitor treatment

Since the profibrotic factors produced by proximal tubular epithelial cells act directly on the renal fibroblasts, the effect of PDGF and CTGF/CCN2 treatment on αSMA production of NRK‐49F cells was also evaluated.

**Figure 6:**
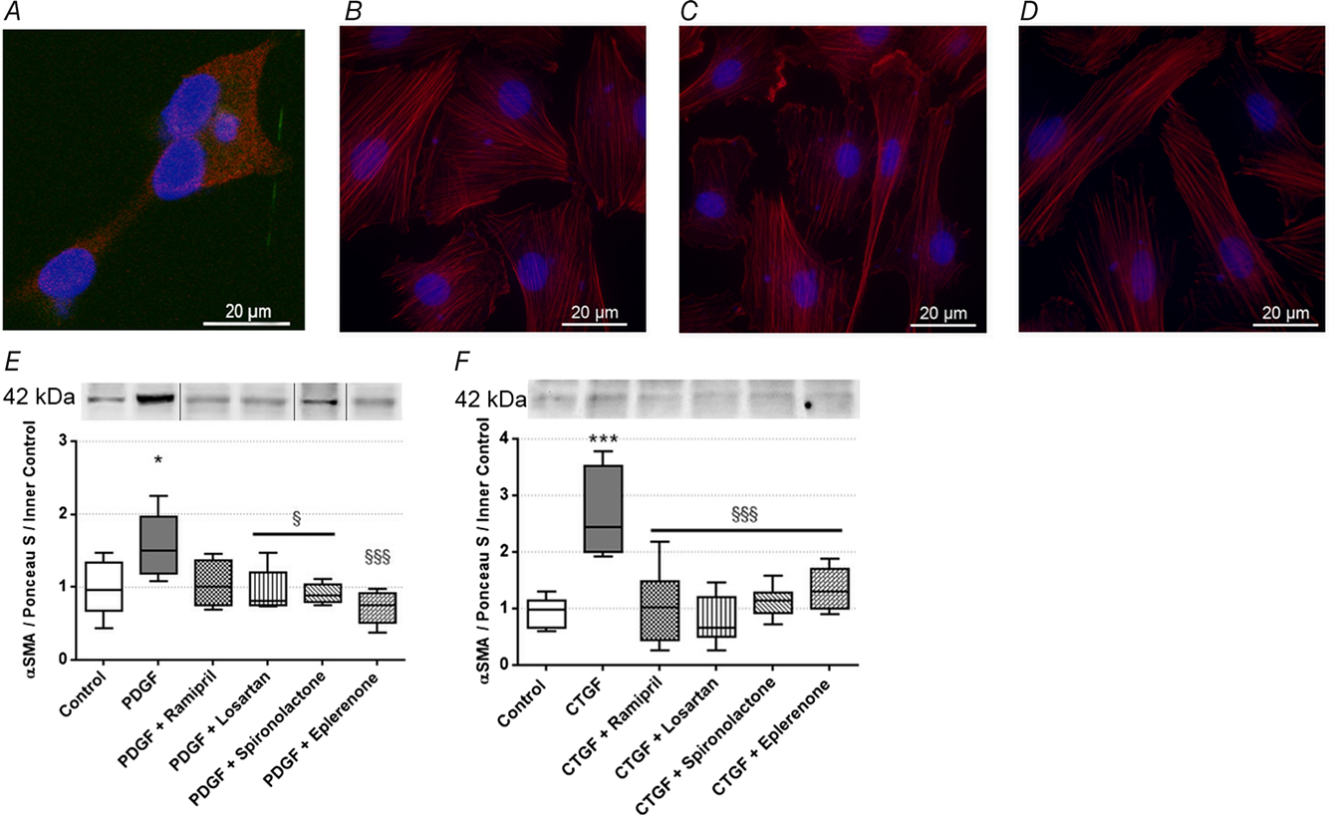
Effect of profibrotic factors and renin‐angiotensin‐aldosterone system inhibitors (RAASi) on normal rat kidney fibroblast (NRK‐49F) cells *A*, representative picture of platelet‐derived growth factor receptor β (PDGFR‐β) stained NRK‐49F cells. 1000× magnification; red, PDGFRβ; blue, nucleus; scale bar = 20 μm. *B*–*D*, representative pictures of phalloidin‐TRITC immunostained NRK‐49F cells (control, *B*) treated with platelet‐derived growth factor (PDGF; *C*) or connective tissue growth factor (CTGF/CCN2; *D*). 1000× magnification; red, F‐actin; blue, nucleus; scale bar = 20 μm. *E* and *F*, alpha‐smooth muscle actin (αSMA) protein levels in NRK‐49F cells treated with PDGF (*E*) or CTGF/CCN2r (*F*) and RAASi. Representative gel image examples shown above the panels. Samples might be from different gels but were derived at the same time and processed in parallel. On each graph values are presented as means ± 95% confidence intervals; *n* = 6 wells/group; one‐way ANOVA followed by Bonferroniʹs multiple‐comparison *post hoc* test; ^*^ *P* < 0.05, ^***^ *P* < 0.001 *vs*. control; ^§^ *P* < 0.05, ^§§§^ *P* < 0.001 *vs*. PDGF or CTGF.

Using fluorescent immunohistochemistry, we firstly proved that NRK‐49F cells express PDGF receptor β (PDGFR‐β) (Fig. 6 *A*), which is essential for the action of both PDGF and CTGF/CCN2. Morphological changes on NRK‐49F cells caused by PDGF and CTGF treatments were visualised by immunostaining of F‐actin with phalloidin‐TRITC. Control cells had a slightly elongated shape and a diffuse actin network of thin actin filaments (Fig. 6 *B*) PDGF and CTGF/CCN2 treatments induced actin cytoskeleton rearrangement in NRK‐49F cells. The elongation of cells was caused by the reorganization of stress fibres along a longitudinal axis, with the formation of F‐actin bundles. Both treatments caused formation of actin‐clumps, especially at the edges of fibroblasts (Fig. 6 *C* and *D*).

Secondly, we showed that PDGFB and CTGF/CCN2 treatments increased αSMA protein levels in renal fibroblasts, indicating fibroproliferative activation and differentiation of NRK‐49F cells. All RAASi hindered these changes, by restoring αSMA to the level of controls (Fig. 6 *E* and *F*).

## Discussion

The frequency of DM is enormously increasing worldwide, thus more and more people are exposed to the risk of developing diabetic complications. DKD is one of the most detrimental consequences of DM regarding patients’ quality of life and survival (Trikkalinou *et al*. 2017). Therefore, there is a great demand for new therapies to slow down the progression of DKD and deepen our understanding of the molecular mechanisms involved. The primary goal of our study was to evaluate the monotherapeutic effect of different RAASi as protection against DM‐induced kidney fibrosis.

DKD is a complex disease, characterised by several pathophysiological changes including glomerular hypertrophy with progressive mesangial expansion, and tubulointerstitial fibrosis. Besides hypertension, hyperglycaemia is also a major contributing factor, and both effects activate local renal RAAS, subsequently increasing intraglomerular pressure, cellular growth, and fibroblast differentiation (Macia‐Heras *et al*. 2012).

RAASi are the standard treatments in the care for hypertensive patients with DM, especially when renal involvement is present (American Diabetes Association, 2018). Their ability to lower blood pressure has been well documented, but they also reduce kidney and glomerular hypertrophy, and renal inflammation without affecting blood pressure (Kobori *et al*. 2013). The antifibrotic potential of angiotensin II receptor 2 agonism (Morrissey & Klahr, 1999), and AT1 antagonism (Gross *et al*. 2004), as well as ACE inhibition (Rubel *et al*. 2014), has been already explored in murine models of unilateral ureteral obstruction and progressive renal fibrosis. Furthermore, RAASi have already been shown to reduce renal fibrosis in DKD (Liao *et al*. 2003; Han *et al*. 2006; Feldman *et al*. 2008), but the complexity of the exact mechanisms is still not fully clarified. To test whether the antifibrotic properties of RAASi are associated with or restricted to their antihypertensive effect, we chose lower treatment dosages avoiding blood pressure changes. In the present study neither diabetes nor RAASi affected blood pressure, which confirms the non‐depressor doses of drugs used in our protocol.

In line with our previous studies, we also observed severe structural and functional kidney damage in diabetic animals, which was improved by all RAASi (Banki *et al*. 2012). According to the literature and our previous results, this renoprotection goes beyond decreasing blood pressure and can be related to other factors like lower oxidative stress (Fiordaliso *et al*. 2006) or O‐GlcNAc levels (Gellai *et al*. 2016).

Overexpression of profibrotic factors such as TGFβ, CTGF/CCN2 and PDGF has been implicated in the development of DKD through the modulation of various signal transduction pathways and renal structural changes (Wang *et al*. 2015). They contribute to the fibrotic process by enabling fibroblasts to differentiate and migrate, which eventually induces the expression of ECM components including collagen type III and MMP9 (Powell *et al*. 1999). In the present study, we demonstrated that monotherapy with various RAASi successfully regulates the hyperglycaemia‐induced fibrotic response and supresses the level of profibrotic factors both *in vivo* and *in vitro*.

To the best of our knowledge, this is the first study to use ECM remodelling biomarkers to test the efficacy of treatment of fibrosis in diabetes. These measurements non‐invasively provide relevant information on renal fibrotic processes and correlate with histological findings. Here we observed that eplerenone could reduce the urinary levels of a biomarker of collagen type III turnover that was highly upregulated in diabetic rats. Aldosterone antagonists restored the biomarker of fibronectin turnover secreted into the supernatant to control levels, which confirmed a decrease in fibronectin turnover in HG‐treated HK‐2 cells. These observations suggest a role for RAASi that involves interfering with the dysregulated remodelling of the interstitial ECM that characterizes fibrosis.

TGFβ1 is one of the three isoforms of TGFβ and it is the most commonly studied profibrotic growth factor. Its relevance has been emphasised in both normal tissue repair (Kane *et al*. 1991) and in the development of several fibrotic processes. It promotes ECM deposition by inducing fibroblasts to differentiate and synthesise fibrotic components (Leask & Abraham, 2004). The expression of *TGFB1* was found to be elevated in human experimental diabetic kidney diseases (Yamamoto *et al*. 1993).

In line with these studies we showed that *Tgfb1/TGFB1* mRNA expression was increased in the kidney of diabetic rats, and also in HG‐treated HK‐2 cells. This increment can be considered an effect of glucotoxicity, rather than hyperglycaemia‐induced hyperosmolarity, since the *TGFB1* level was not increased by the isosmotic control mannitol treatment.

The connection between TGFβ and RAAS inhibition in STZ‐diabetes has been discussed in only a few studies so far. These showed that some RAASi attenuate renal *TGFβ1* expression in STZ‐diabetic rats, although in these studies valsartan (Huang *et al*. 2011), and aliskiren (Feldman *et al*. 2008) treatments were initiated from the beginning of the diabetes and the drugs were administered directly into the renal cortex or subcutaneously, but not orally. Other studies in which RAASi successfully affected TGFβ1 levels have been conducted on other kidney disease models such as the subtotal nephrectomy model (Piecha *et al*. 2008), and used different type of RAASi (Liao *et al*. 2003). Interestingly, in our study, *per os* RAASi had no effect on *Tgfb1* expression either in the diabetic rat kidney, or in HK‐2 cells. Therefore, it appears that *Tgfb1* reduction is not one of the antifibrotic mechanisms by which RAASi act in the advanced stage of DKD in STZ rats.

The expression of other profibrotic factors like PDGF and CTGF can be activated through TGFβ‐dependent and ‐independent pathways (Yang *et al*. 2009). Both factors can act through PDGFR‐β (Makino *et al*. 2017), which is widely distributed throughout the cytoplasm of fibroblasts (Huang & Huang, 1988), as we also detected in our study.

PDGF consists of homo‐ and heterodimers and PDGF‐BB is crucial for production and maintenance of renal structures and ECM (Ostendorf *et al*. 2012). PDGF is also involved in structural alterations of DKD. Di Paolo *et al*. (1996) demonstrated that HG induced a persistent increase in *PDGFB* expression, thus stimulating fibroblast proliferation, collagen and ECM production. Ramipril, eplerenon, irbesartan and spironolactone decreased PDGFB in experimental hepatic (Li *et al*. 2005), myocardial (Nishioka *et al*. 2007) and atrial fibrosis (Yang *et al*. 2013) and in cyclosporine nephrotoxicity (Macunluoglu *et al*. 2008). To the best of our knowledge the effect of RAASi on PDGFB has not yet been investigated in DKD.

CTGF/CCN2 is a matricellular protein that promotes angiogenesis, inflammation, tissue repair and fibrosis (Lipson *et al*. 2012). Overexpression of *CTGF* is now widely accepted as a key marker of fibrotic activity. In diabetic kidneys the expression level of *CTGF* in the kidney is increased (Wang *et al*. 2001), and high glucose levels were also identified as an *in vitro* stimulus (Murphy *et al*. 1999). Losartan and spironolactone have been shown to decrease CTGF/CCN2 in human (Andersen *et al*. 2005) and experimental DKD (Han *et al*. 2006).

In agreement with earlier reports both in the diabetic kidneys (Floege *et al*. 2008), as well as in HG‐cultured HK‐2 cells (Mesarosova *et al*. 2017), we found higher *PDGFB* and *CTGF* expression in our rat model. RAASi decreased the production of both growth factors and also diminished their expression when administered to renal proximal tubular cells, suggesting that these drugs directly act on proximal tubules by inhibiting HG‐induced upregulation of the profibrotic *PDGFB* and *CTGF* genes, but surprisingly not the *TGFB1* gene.

As we treated NRK fibroblasts with either PDGF or CTGF/CCN2, morphological changes showed actin cytoskeleton rearrangement. Similar transformations have been described in human fibroblasts (Hedberg *et al*. 1993), and smooth muscle cells (Kuzuya *et al*. 2004) due to PDGF treatment; and in podocytes (Fuchshofer *et al*. 2011) after CTGF/ CCN2 administration. Furthermore, in our experiments the production of αSMA also increased, indicating myofibroblast differentiation. We showed for the first time that all the RAASi tested successfully decreased this fibrotic transformation, indicating that these cells might be one of the primary targets of RAASi.

MMP2 is an endopeptidase responsible for the degradation of the major components of ECM proteins, and thus it has an important role in senescence and fibrosis. The renal expression of *Mmp2* was shown to increase as a compensatory mechanism in STZ‐induced DKD (Takamiya *et al*. 2013), as we also observed in the present study. In renal cortex of diabetic rats, higher *Mmp2* expression was decreased by RAASi, most successfully by aldosterone ant agonists. TIMP1 is an endogenous inhibitor of MMPs which influences ECM integrity and the tissue microenvironment. Its expression was also increased in the kidneys of diabetic rats, indicating that fibrosis increased because ECM degradation was restricted, and RAASi treatments prevented TIMP1 production.

In summary, we describe here a novel molecular mechanism by which RAASi are effective in the prevention of hyperglycaemia‐induced fibrosis. We showed that RAASi directly target renal proximal tubular cells by reducing PDGFB and CTGF, but not TGFβ1 production, and also inhibit αSMA accumulation in kidney fibroblasts. Ours is the first study to compare different RAASi and suggest that monotherapy with aldosterone antagonists might be as effective as, or even more effective than ACE inhibitors or angiotensin II receptor blockers in preventing the progression of renal fibrosis in DKD.

In conclusion, the present study demonstrates that RAASi drugs in monotherapy are effective at supporting kidney function and preventing renal fibrosis in STZ‐induced DKD. We suggest that alteration of profibrotic factor production and fibroblast differentiation might serve as an additional target of RAASi and could facilitate their monotherapeutic application in the clinical management of renal fibrosis. A better understanding of these targets could facilitate the development of novel therapeutic strategies translated into clinical applications, but well‐controlled human clinical trials are needed to confirm these suggestions and to evaluate of the risk/benefit ratio, especially of aldosterone antagonists.

## Additional information

### Competing interests

N.S. and F.G. are full‐time employees at Nordic Bioscience. All authors disclose that they have no conflict of interest to declare.

### Author contributions

The experiments were performed in the laboratory of the 1st Department of Paediatrics, Semmelweis University, Budapest, Hungary. S.K., A.M., J.H., D.B.B., E.Sz., N.S. and F.G. performed the acquisition, analysis and interpretation of data. S.K. and A.M. drafted the work. J.H. contributed to the design of the work. L.L., A.F. and W.L. contributed to the conception of the work and revised it critically for important intellectual content. All persons designated as authors qualify for authorship, and all those who qualify for authorship are listed. All authors approved the final version of the manuscript and agree to be accountable for all aspects of the work in ensuring that questions related to the accuracy or integrity of any part of the work are appropriately investigated and resolved.

### Funding

This work was funded by the grants of OTKA‐K112629‐K116928‐FK124491‐NN‐11460, VKE‐2017‐00006, EEMOFAKT‐2017, EFOP‐3.6.3‐VEKOP‐16‐2017‐00009, and was also supported by an MTA‐SE “Lendület” Research Grant LP008/2017.

**Translational perspective**

Diabetic kidney disease (DKD) is the leading cause of end stage renal disease, where the increased activation of the renin‐angiotensin‐aldosterone system (RAAS) contributes to renal fibrosis. We investigated the effect of hyperglycaemia on the development of fibrosis and its signal‐transduction pathway *in vivo* and *in vitro*, and examined the possible antifibrotic effect of RAAS inhibitors (RAASi). In the present study we report that in type 1 diabetic rats renal interstitial fibrosis was ameliorated, while elevated profibrotic factor levels were decreased by RAASi. Hyperglycaemia‐induced profibrotic factor production in proximal tubular epithelial cells increases fibroblast activation, both of which effects can be prevented with RAASi treatment. These results can help establish new therapeutic possibilities for the treatment of DKD and facilitate the monotherapeutic application of aldosterone antagonists. This animal study provides a basis for future clinical trials of the antifibrotic potential of RAASi treatment. Such studies may further reveal the mechanisms underlying the pleiotropic renoprotective effect of RAASi.
